# Angiogenesis and tissue formation driven by an arteriovenous loop in the mouse

**DOI:** 10.1038/s41598-019-46571-4

**Published:** 2019-07-19

**Authors:** Richard Wong, Roberto Donno, Christopher Y. Leon-Valdivieso, Urmas Roostalu, Brian Derby, Nicola Tirelli, Jason K. Wong

**Affiliations:** 1 Division of Cell Matrix and Regenerative Medicine, Faculty of Biology, Medicine and Health, Manchester Academic Health Science Centre, University of Manchester, Manchester, M13 9PT UK; 2 Laboratory of Polymers and Biomaterials, Fondazione Istituto Italiano di Tecnologia, Via Morego 30, 16163 Genova, Italy; 3 School of Materials, University of Manchester, Manchester, M13 9PL UK; 4 Present Address: Roberval Laboratory for Mechanics, Sorbonne Universités, Université de Technologie de Compiègne, Rue du Dr. Schweitzer, 60200 Compiègne, France; 5 Gubra, Horsholm, Denmark; 6 Division of Pharmacy & Optometry, School of Health Science, University of Manchester, Manchester, M13 9PT United Kingdom; 7 Department of Burns and Plastic Surgery, Manchester University Foundation Trust, Manchester Academic Health Science Centre, Wythenshawe Hospital, Southmoor Road, Manchester, M23 9LT UK

**Keywords:** Regenerative medicine, Angiogenesis

## Abstract

The rapid vascularisation of biomaterials and artificial tissues is a key determinant for their *in vivo* viability and ultimately for their integration in a host; therefore promoting angiogenesis and maintaining the newly formed vascular beds has become a major goal of tissue engineering. The arteriovenous loop (AVL) has been an extensively studied platform which integrates microsurgery with cells scaffolds and growth factors to form neotissues. Most AVL studies to date are limited to larger animal models, which are surgically easier to perform, but have inherent limits for the understanding and interrogation of the underlying *in vivo* mechanisms due the paucity of transgenic models. Here, we demonstrate for the first time in a mouse model the utility of the AVL in the *de novo* production of vascularized tissue. We also present the combined use of the model with 3D printed chambers, which allow us to dictate size and shape of the tissues formed. This novel platform will allow for an understanding of the fundamental mechanisms involved in tissue generation *de novo*.

## Introduction

The viability of replacement tissues and organs lost through trauma, infection, malignancy or congenital abnormality depends on the presence of a functional circulatory system with a capillary network providing nutrient and gas exchange. One of the challenges of tissue engineering is to develop rapidly perfusable vascularized tissue by mimicking natural vascular architecture and rebuilding microvascular networks, that is surgically compatible^1–3^. One potential solution is the pre-fabrication of scaffolds with cells, a vascular network and large vessels. The arteriovenous shunt loop (AVL), an anastomosis between an artery and a vein that shunts arterial blood into the vein, has been shown to spontaneously generate blood vessels *in vivo*^1^. Along with angiogenesis, the AVL has also been shown to stimulate *de novo* tissue formation when enclosed within an artificial chamber^4^ filled with poly(lactide-co-glycolide) (PLGA), Matrigel or fibrin^5^ as extracellular matrix mimicking materials. Angiogenesis and tissue development can be further enhanced with addition of angiogenic growth factors like vascular endothelial growth factor (VEGF) or basic fibroblast growth factor (bFGF)^6^. It is thought that following surgical creation of the AVL, cells like pericytes, macrophages, fibroblasts and neutrophils, migrate/differentiate into the peripheral tissues three days after loop implantation creating a hypoxic gradient between regions adjacent to the loop and the matrix boundary^7^. This hypoxic environment exists in the first week following implantation of an AVL^8^, but disappears once an intrinsic vascular network establishes after 4–6 weeks^9^. It is unclear whether hypoxia is the main driver for angiogenesis but it has been shown that shear stress from arterially pressured blood within vein vessels is important^10–12^. Thus, the precise mechanisms that underlie this vascular assembly are uncertain.

Most investigations of the AVL have been limited to the rat model with a few studies in larger models such as rabbits^13^, goats^14^ and sheep^15,16^. In this paper, we demonstrate for the first time, the creation of an AVL in the mouse with dictation of size and shape of the constructs using 3D printed scaffolds. The benefits of establishing the AVL in the mouse is that we may take advantage of the vast transgenic mouse libraries that are already established. Thus, allowing us to investigate candidate pathways and further identify important regulators of angiogenesis and tissue formation in a wound healing and tissue engineering context.

## Results

### Macroscopic appearance, viability and weight

**Figure 1:**
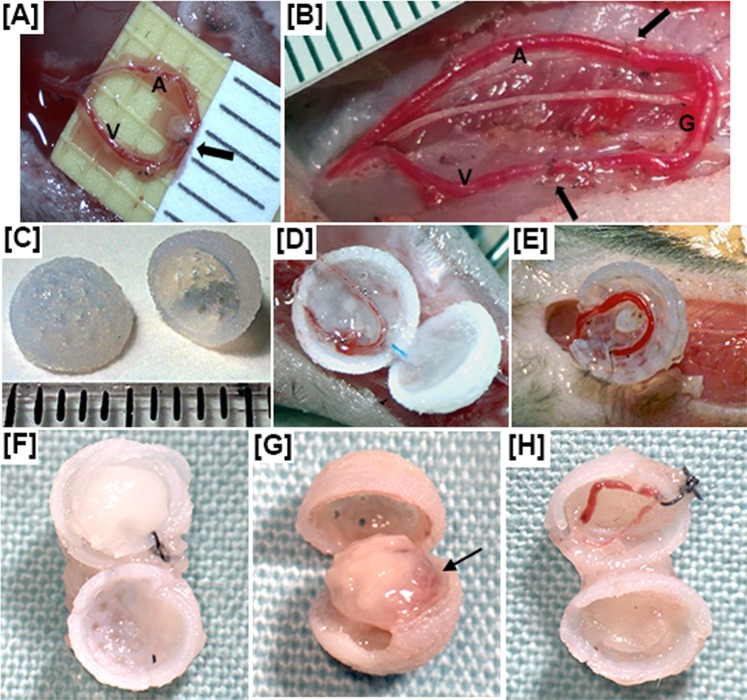
We show the AVL in the mouse (**A**) compared with the AVL in the rat (**B**). The artery is denoted by “A”, vein by “V” and graft by “G”; arrows denote site of anastomosis. There is no graft for the mouse AVL. (**C**) 3D-printed sphere chambers for mouse AVL. For (**A**–**C**) each division on the scale represents 1 mm. (**D**) Mouse AVL in the chambers containing fibrin matrix. (**E**) AVL pedicle with no flow as a control. We also show the contents after 28 days for a control (**F**) that contained fibrin matrix only and no AVL; (**G**) a mouse AVL within fibrin matrix (arrow denotes region of possible blood vessel development); and (**H**) a mouse AVL pedicle with no flow as a control.

We collected chambers and visually examined the contents after 4, 21 and 28 days (Supplementary Fig. S1). There were some remnants of blood from the AVL procedure after 4 days, but this blood was no longer apparent at 21–28 days. At 28 days, controls without AVL retained an appearance similar to day 0 (Fig. 1F), AVL chambers contained soft tissue in a roughly spherical shape, with regions of possible blood vessel development where the AVL was placed (Fig. 1G), and finally, AVL controls without flow showed reduced fibrin matrix but little tissue or blood vessel formation had occurred to replace the matrix (Fig. 1H). Instead, the chambers were difficult to open and a fibrous scar-like encapsulating layer, which was found on the surface of all spheres at 28 days, was found inside of these controls as well.

**Figure 2:**
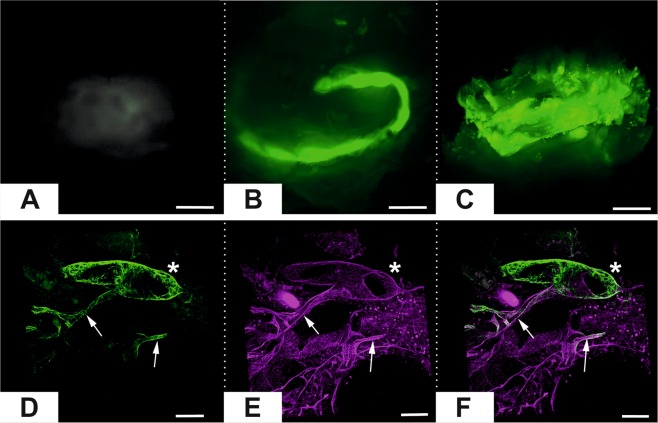
*Top*. Light sheet imaging fluorescent microscopy of the contents of mouse chambers. (**A**) Contents of chamber with no AVL control after 7 days. (**B**) AVL after 7 days. (**C**) AVL after 28 days. Scale bar 1 mm. *Bottom*. Confocal imaging of day 28 AVL tissue. (**D**) FITC Dextran perfusate of vascular channels in chamber. (**E**) CD31 labelling of endothelium. (**F**) Merged image of colocalised perfusate and endothelial channels. Scale Bar 100 µm.

**Figure 3:**
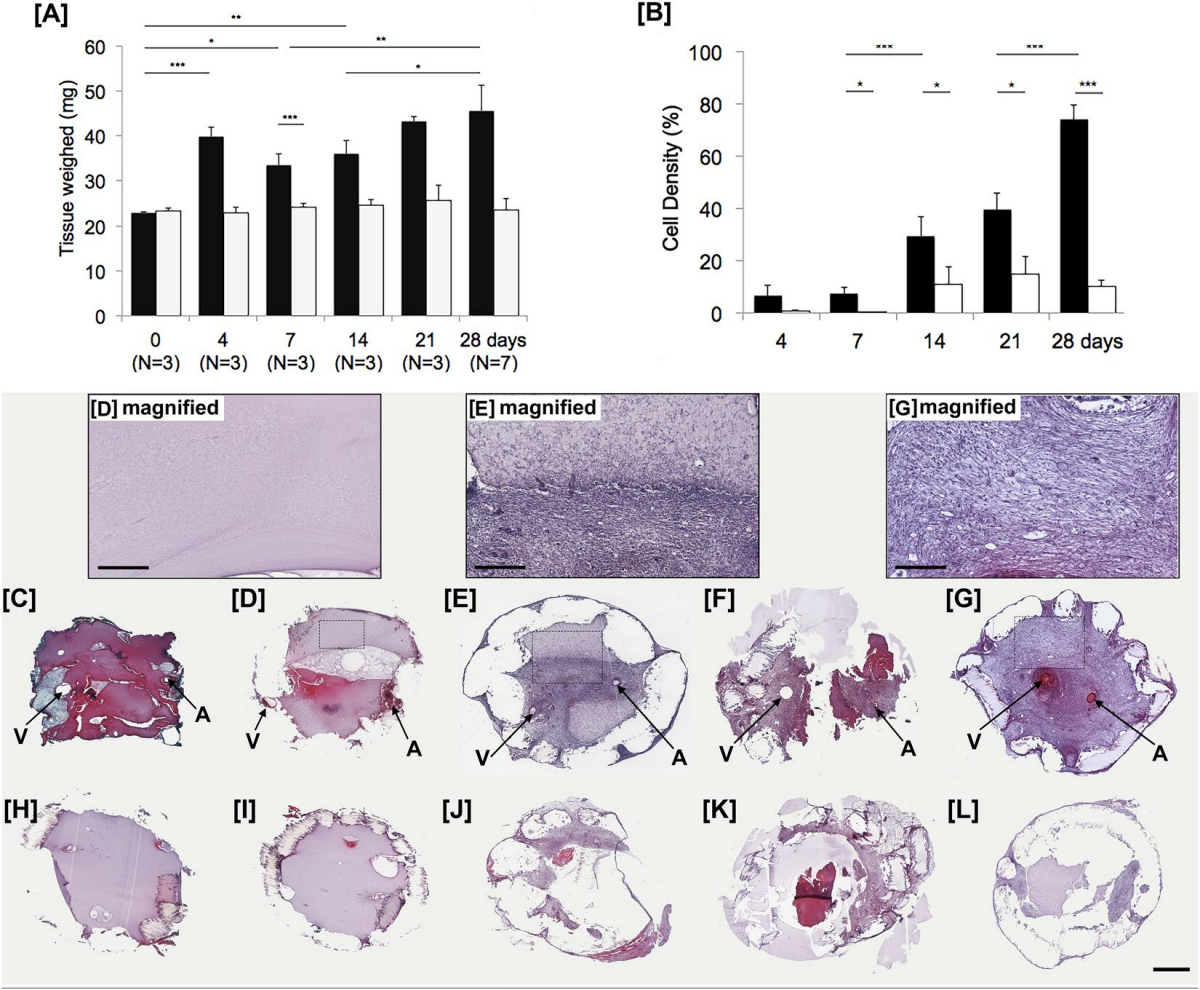
Analysis of the chambers containing AVL and no AVL over time. (**A**) The weight of contents within chambers of AVL (black) vs. no AVL controls (white). (**B**) The cell density (expressed as a percentage of the total chamber area, mean ± SD) over time. ***P < 0.001, *P < 0.05. The cell density was obtained by analyzing H&E sections in (**C**–**G**) AVL compared to (**H**–**L**) no AVL control chamber at day 4 (**C**,**H**) 7 (**D**,**I**) 14 (**E**,**J**) 21 (**F**,**K**) and 28 (**G**,**L**). Magnified views are included for AVL time-points at days 7, 14 and 28. Arrows V = vein and A = artery. Scale bar = 1 mm. Scale bar within magnified view = 300 µm.

Light sheet fluorescent imaging demonstrated that there was no visible perfusion of the chambers without AVLs but perfusion of the AVL of tissues in the chamber was evident in all animals (N = 3). At 1 week the loop was easily defined but by 4 weeks the loop was hard to define due to the fluorescence from the whole tissue (Fig. 2A–C). Confocal microscopy at higher resolution confirmed that the vascularised tissue was patent as the large and small vessels were perfused with FITC dextran after 28 days (Fig. 2D). This indicates that the vascular channels formed from the AVL, function for blood delivery. H&E sections of the chambers showed the fibrin matrix that is visible at 4 days and 7 days is gradually colonized by cells between 14 and 28 days (Fig. 3C–G), with less cells observed in controls (Fig. 3H–L). We measured the density of cells (Fig. 3B) found in the AVL samples, and significant increases from day 7 (7.3 ± 2.4%) to day 14 (29.5 ± 7.3%) and from day 21 (39.5 ± 6.2%) to day 28 (73.9 ± 5.6%) were observed. Whereas in the controls without an AVL, we observed significantly less cells with a maximum of 15.0 ± 6.5% cell area at day 21. These findings reflected the change in weight of the content inside the chambers over time (Fig. 3A). We found that the AVL significantly increased the average weight from 22.9 ± 0.3 mg at day 0 to 45.6 ± 5.7 mg at 28 days, while the controls without AVL did not significantly change (23.4 ± 0.6 mg to 23.5 ± 2.5 mg). No flow controls significantly increased to a lesser degree from 23.4 ± 0.7 mg at day 0 to 26.9 ± 1.4 mg at 28 days. In the AVL cohorts, the average weight increased significantly from 0 (22.9 ± 0.3 mg) to 4 days (39.8 ± 2.2 mg), followed by a decrease in the weight at 7 days (33.5 ± 2.6 mg), followed by a significant increase from 14 to 28 days (36.0 ± 2.9 mg to 45.6 ± 5.7 mg).

### Angiogenesis

**Figure 4:**
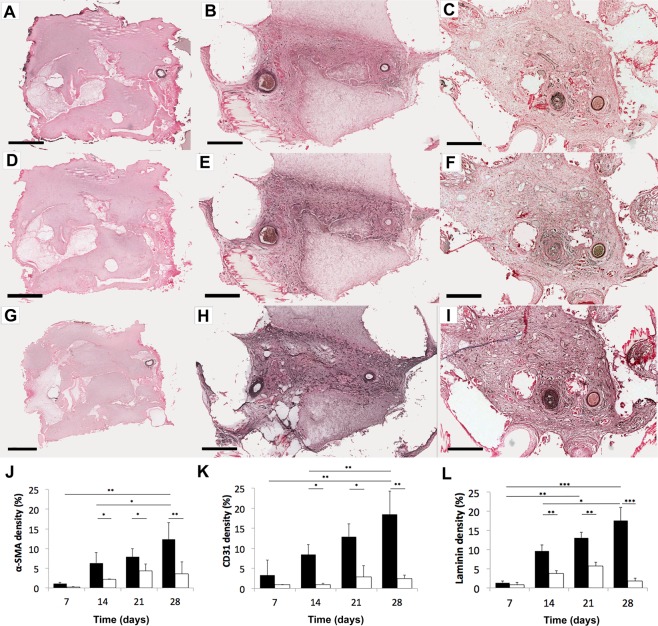
Sections from AVL chambers for blood vessel markers. (**A**–**C**) α-SMA (blood vessel wall), (**D**–**F**) CD31 (endothelial cell), (**G**–**I**) laminin (basement membrane) were stained at day 7 (left column), day 14 (middle), day 28 (right). Increasing numbers of blood vessels (dark brown structures) appear with increasing time. Scale bar = 500 µm. The areas of staining for (**J**) α-SMA (K) CD31 (**L**) laminin were then measured and expressed as a percentage of the total chamber area, mean ± SD in AVL (black) compared to no AVL controls (white) over time. ***P < 0.0001, **P < 0.001, *P < 0.05. Larger magnifications can be found in Supplementary Fig. S5A–F.

From immuno-staining for α-SMA (vessel walls), CD31 (endothelial cell adhesion), and laminin (basement membrane) (Fig. 4), we observe a significantly greater level of blood vessels in the AVL compared to no AVL controls from day 14 onwards (Table 1). At day 7 the AVL samples showed a higher level of markers, but the difference with the controls was not statistically significant. We also see less blood vessel development in no AVL controls (Supplementary Fig. S2) and ligated no-flow AVL controls at day 28 (Supplementary Fig. S3). Observing blood vessel development within AVL samples, we found blood vessels to significantly increase (approximately double) between day 14 and day 28: α-SMA (P = 0.0392), CD31 (P = 0.0062) and laminin (P = 0.048). We counted the number of individual blood vessel structures (stained by CD31) in centre histological sections of chambers and observed that no blood vessels formed on day 7 but was observed from day 14 onwards in the AVL cohort (Supplementary Fig. S4). Furthermore, blood vessels were significantly greater (mean 486 vs 301, P = 0.0112) in the venous side of the AVL compared to the arterial side on day 28. Blood vessels were found only on day 28 in no AVL controls (mean 42 vessels), and these had ingressed from the outside through the pores of the chambers.

**Table 1:** Markers for blood vessels (expressed as a percentage of the total chamber area, mean ± SD) in AVL (above) and no AVL controls (below).

	7 days	14 days	21 days	28 days
**α-SMA (%)**	1.06 ± 0.38 (0.27 ± 0.12)	6.32 ± 2.64 (2.21 ± 0.07)	7.89 ± 2.11 (4.35 ± 1.77)	12.35 ± 4.29 (3.58 ± 3.02)
p-value (AVL vs. no AVL)	0.1029	0.0208	0.0231	0.0037
**CD31 (%)**	3.27 ± 3.75 (0.90 ± 0.35)	8.42 ± 2.52 (0.91 ± 0.35)	12.82 ± 3.27 (2.86 ± 2.78)	18.45 ± 5.83 (2.49 ± 0.85)
p-value (AVL vs. no AVL)	0.3159	0.0041	0.0219	0.0002
**Laminin (%)**	1.37 ± 0.44 (0.85 ± 0.58)	9.60 ± 1.63 (3.76 ± 0.74)	13.02 ± 1.47 (5.71 ± 1.00)	17.55 ± 3.43 (1.85 ± 0.68)
p-value (AVL vs. no AVL)	0.2815	0.0099	0.0035	0.0001

P-values are given below for t-test comparisons between AVL and controls.

### Cell proliferation and tissue formation

**Figure 5:**
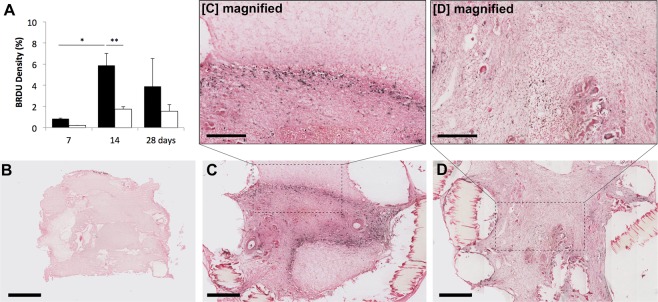
(**A**) Proliferative cells are labelled by BrdU staining and appear black on images. These are then expressed as a percentage of the total chamber area (mean ± SD) for AVL (black bars) compared to no AVL controls (white) over time. Images of AVL chambers are shown for (**B**) day 7, (**C**) day 14 and (**D**) day 28. Scale bar = 500 µm. Magnified views are included for AVL at days 14 and 28, which indicate that proliferative cells are mostly found at 14 days at the boundary between newly formed tissue and the fibrin matrix. Scale bar within magnified view = 300 µm.

A high number of proliferative cells was observed at 14 days (Fig. 5) with most of the proliferation occurring at the border between the newly formed tissue and the fibrin matrix. The tissue that has formed may have arisen from an advancing wave of proliferative cells from the AVL. While very little proliferation was found in the AVL at day 7 (0.85 ± 0.05%), a significant (P = 0.0234) number was found at day 14 (5.87 ± 1.2%) followed by an insignificant (P = 0.3757) decrease at day 28 (3.90 ± 2.6%). This level of proliferation was not observed in the no-AVL controls and was significantly different at 14 days (1.75 ± 0.23%, P = 0.0038). Laminin staining (Fig. 4G–I) suggests that there is an abundant level of basement membrane and extracellular matrix formed over time.

**Figure 6:**
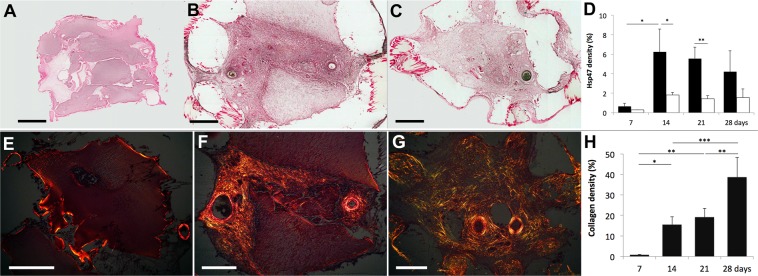
Hsp47 staining for collagen synthesis chaperone is shown for (**A**) Day 7, (**B**) day 14 and (**C**) day 28. (**D**) We found the level (percentage of the total chamber area, mean ± SD) of Hsp47 to be significantly greater in AVL (black) compared to no AVL controls (white) at day 14 and 21. Sample images of picrosirius staining for collagen bundles are shown for (**E**) day 7, (**F**) day 14 and (**G**) day 28. All samples were measured at 0° and 90° rotations, and the areas of collagen birefringence were calculated as a percentage of the total chamber area (mean ± SD). We find that the collagen increases with time, and the greatest increases of collagen were from day 7 to day 14, and day 21 to day 28. Scale bar = 500 µm. 900 × 327 mm (180 × 180 DPI).

Extracellular matrix formation was reflected when we measured for the presence of Hsp47, a chaperone of collagen synthesis (Fig. 6A–D) and for collagen fibres with picrosirius staining (Fig. 6E–H). The density of Hsp47 staining in the AVL chambers was found to be greatest at 14 days (6.25 ± 2.3%) and significantly greater than no-AVL controls (1.83 ± 0.24%). There was a decreasing trend of collagen synthesis at 21 days (5.56 ± 1.2%) and 28 days (4.21 ± 2.1%); whereas Hsp47 staining remained low in the no-AVL controls. The density of collagen fibres significantly increased with time in AVL chambers with 0.78 ± 0.16% at day 7, 15.5 ± 3.9% at day 14, 19.1 ± 4.3% at day 21 and 38.5 ± 9.7% at day 28.

## Discussion

The AV shunt loop model has been successful used to promote angiogenesis to vascularize bone^17,18^, muscle^19,20^, liver^21,22^, functioning cardiac tissue^23,24^, fat^25,26^, pancreas^27,28^ and thymus tissue with de novo T-cell production^29^. Despite this promise, there is a limit to the new vessel formation and the size of tissue generated^30,31^. In a clinical trial, three out of four patients failed to generate sufficient volumes of fat for breast reconstruction (Neopec)^32,33^, suggesting the clinical translation of this technology has some fundamental unknowns that require dissection. Thus, if we could extend our understanding of the mechanism underlying angiogenesis, we may be able to generate clinically relevant volumes of tissue for therapy. Most mouse models described in the literature are limited to the flow-through model^34–36,37^ but the AVL model has been shown to produce the most vascularized tissue in comparison^38^. It is important to the field of tissue engineering that a mouse model for the AVL is developed to characterize the mechanisms that underlie the angiogenesis phenomenon.

Mouse models of microvascular research pose a particular technical challenge, and requires considerable microsurgical experience to establish. However, the advent of supermicrosurgery and the growing experience of microsurgery research laboratories mean that the reliability of these procedures is increasing, with numerous examples of how these techniques can be adopted. Hind limb^39^ and facial^40^ transplantation in mice is now achievable, and the vessels repaired in AVL are of a similar calibre. We found in our hands the intrastent technique described by Narushima *et al*. works best^41^ however techniques such as the cuff technique is also a reliable option^42^.

We have demonstrated that the mouse AVL model is achievable and that the vasculature remains patent, and that it promotes spontaneous angiogenesis to generate vascularized tissue. We observed that very little angiogenesis or tissue generation occurs at early time-points (between 4–7 days), but increased dramatically by 14 days and entirely fills the chamber space replacing the fibrin matrix with cells and extracellular matrix by 28 days. The corresponding weight measurements support this increase in tissue over time. Some vessel in-growth into the chamber occurs via its pores at 28 days, but as reported in rats^9^, the majority of the newly formed blood vessels stems from the AVL itself. Our data also showed that a greater number of vessels had formed on the venous side of AVL than the arterial side (Supplementary Fig. S4), supporting the notion that arterially-pressured blood within thinner vein walls may trigger greater angiogenesis^10–12^. We also observed vessel in-growth via the pores of the chamber in the no AVL and ligated AVL controls at day 28 but we do not see the same extent of angiogenesis or tissue formation (Supplementary Figs S2 and S3). It may be that pores in the 3D printed chamber allow for wound-healing molecules and other substrates to diffuse from the surrounding environment into the chamber^43,44^ or alternatively, allows movement or flow out of the scaffold hence promoting a gradient of growth away from the loop. Without pores, there may be an intrinsic pressure within the chamber that prevents cell growth towards to the periphery, thus reducing the level of tissue generated^43^. Further experiments are required to explain the role of scaffold pores in the AVL model. At 14 days, we see the tissue generated encroach into the matrix from the AVL with an expanding wave of proliferating cells towards the periphery in a radial fashion. This is most likely due to gradients of chemoattractants from the AVL^44^, which can be appreciated completely in the mouse model because of the scale. Analysis of the collagen bundles using picrosirius staining and rotated polarized light, together with the measurements of Hsp47 and laminin suggests that the tissue produced by the AVL increasingly resembles extracellular matrix over time. Although the abundance of collagen increased proportionally with time, synthesis of the collagen occurred the most at 2 weeks. Red and green birefringence can be used to determine collagen type I and type III^41^ but its specificity is questionable^45,46^. The use of picrosirius red has been more accurately used to determine fibre thickness, with green birefringence relating to thinner fibres, and red to thicker^46^. Using a similar technique, we observed at earlier time-points, the collagen to be predominantly thick fibred but there was a significant increase in thin bundles at 28 days. This is unlike scar or capsular fibrosis, which has a distinct polarity to its organization^47^. Electron microscopy and collagen type I/type III immunohistochemistry may allow us to determine further the composition of this collagen matrix. Tissue loss as a consequence of trauma, infection, malignancy, or congenital abnormality is a significant clinical problem. Surgical solutions include autologous tissue reconstruction in the form of local and free tissue transfer introduces donor site morbidity^48–51^, surgical risk^52^ and has significant consequences in cases of flap thrombosis^53^, infection^54^ and total failure^55^. Tissue engineering solutions, such as allogeneic scaffolds require blood vessel formation to insure its survival and incorporation. Thus, promoting endogenous vascularization processes to prevascularize scaffolds prior to cell injection and implantation are essential to building replacement tissues and organs. The processes and mechanisms that underlie new blood vessel formation are currently unclear. By developing the AVL in the mouse, it provides a platform through genetic manipulation to investigate which mechanisms are important to angiogenesis and tissue formation.

## Methods

### Surgical procedures

All animal procedures were approved by the Local Ethical Committee at the University of Manchester and complied with British Home Office regulations on the care and use of Laboratory animals under PPL 70/8686. 8–10 week old C57/Bl male mice (Harlan Laboratories) were anaesthetized by isoflurane (Abbot Laboratories Ltd, UK) (induction at 4 L/min oxygen with 4% isoflurane, maintained at 2 L/min oxygen with 2% isoflurane during the rest of the procedure). Mice were immobilized on their posterior and the left hind limb was shaved up to the arcuate line. Under an operating microscope (Leica MZ7.5, Leica Microsystems, Switzerland), the femoral artery and vein between the junction of the tibial veins to the inguinal ligament were carefully exposed from surrounding tissues with curved microscissors (SDC-15, Mercian Surgical, UK) and forceps (Dumont #5/45, Fine Science Tools, Germany). Vessels feeding into the vein were cauterized (Bovie, USA) or ligated with 11–0 sutures (Ethilon, Ethicon, UK). The distal ends of the femoral artery and vein were isolated, clamped (Acland B-1 00396V, Mercian Surgical, UK). A 5–0 prolene suture was used as an intravascular stent (Ethicon, UK) to allow anastomosis with 12–0 sutures (S&T, Mercian UK) to create an arteriovenous shunt loop (AVL) (Fig. 1A and Supplementary Video 1). Our patency rates were 100% for the data presented in this study. Patency of flow through the loop was checked by performing an Acland test (S Video 2). No anti-coagulants were used.

The AVL was placed into the 3D printed chamber containing fibrin matrix, and the semi-spheres were sutured together (Fig. 1D). This single sphere was secured into space created in the left inguinal region with 8–0 sutures (Ethilon, B.Braun, Germany), and the skin was closed with two layers of 8–0 sutures. A control sphere that contained only fibrin matrix and no AVL was secured into space created in the right inguinal region. Further controls contained fibrin matrix and AVL but ligated to prevent flow were also performed (Fig. 1E). The ligated AVL controls increased the overall time of surgery and could not be practically performed in the contra-inguinal area of experimental mice; and additional animals were required to create ligated AVL controls (Table 2). Analysis of 28 day timepoint ligated AVL controls (Supplementary Fig. S3) suggest these controls were comparable to no AVL controls (Supplementary Fig. S2), and ligated AVLs were not performed for the other timepoints.

**Table 2:** The following table shows the number of mice reserved for preliminary and macroscopic analysis (denoted as PRE); histology and immunohistochemistry sectioning (denoted as IHC); FITC-Dextran perfusion and light-sheet fluorescent microscopy (denoted as LSFM); and whole-mount IHC (denoted as WM-IHC).

Timepoint (days)	0	4	7	14	21	28
AVL	—	PRE = 3	—	—	PRE = 3	PRE = 3
	IHC = 3	IHC = 3	IHC = 3	IHC = 3	IHC = 3	IHC = 4
	—	—	LSFM = 3	—	—	LSFM = 3
	—	—	WM-IHC = 3	—	—	WM-IHC = 3
No AVL control	Control chambers were placed in the contra-inguinal region of experimental mice. The number of control chambers will mirror that of AVL chambers.					
AVL (ligated no-flow control)	—	—	—	—	—	IHC = 3

Analgesia (0.1 mg/kg, Vetergesic, Alstoe Animal Health) was administered and animals were allowed to recover with 2 L/min oxygen. All animals underwent the surgical procedure well without any major complications such as extrusion of the chambers, infections or hematomas. The mass of chambers were weighed at harvesting. Vascularity of the AVLs was inspected at time of harvesting and confirmed during histological examination – patency of the vasculature determined by absence of organised thrombosis in the vascular channels on histological review. All AVL constructs analysed were found to be patent. All AVL chambers were found to have vessel formation. Intraperitoneal injections (1 mL per 100 g body weight) of bromodeoxyuridine (BrdU) (#RPN201, GE Healthcare, UK) were given to animals 4–6 h prior to harvesting AVL.

### Chamber production

Chambers were designed using 3D CAD software (Autodesk Inventor 2015, student edition) as 5 mm diameter semi-spheres with 0.5 mm thick walls. 1 mm diameter pores were distributed uniformly on the surface. These were printed with a biocompatible acrylic/acrylate modelling material (Veroblue RGD840, Stratasys) in an Objet30 3D printer (Stratasys, USA). Following print completion, support material (FullCure 705, Stratasys) was removed by immersing in 1% sodium hydroxide solution for 3 h. Chambers were washed in distilled water and sterilized by exposure to UV light for 30 min (Fig. 1C).

### Fibrin matrix preparation

Bovine fibrinogen (F8630, MW: 340 kDa), bovine thrombin (T9549, ≥1500 NIH units/mg protein) and HEPES buffer were purchased from Sigma (Gillingham, UK). CaCl_2_ and NaCl were purchased from Fisher (Loughborough, UK). Stock solutions of fibrinogen, thrombin and CaCl_2_ were prepared at a concentration of 75 mg/mL, 6 U/ml and 120 mM, respectively, in HEPES-buffered saline (HBS: 20 mM HEPES; 150 mM NaCl, pH = 7.4). Complete dissolution of fibrinogen is achieved after 2 h of incubation at 37 °C under shaking (150 rpm, Heidolph Titramax 1000). The precursor solutions were sterile-filtered (0.22 µm PES filters) and mixed in appropriate volume ratios to achieve the final concentrations of 25 mg/mL, 1 U/mL and 20 mM, respectively for fibrinogen, thrombin and CaCl_2_. Gels were prepared by mixing CaCl_2_ solution with thrombin solution in equal volumes, obtaining a CaCl_2_-thrombin solution. The CaCl_2_-thrombin solution, HBS buffer and fibrinogen solution were mixed in a volume ratio equal to 1:1:1. For example, to prepare 600 µL of fibrin gel precursor solution: 100 µL of CaCl_2_ stock solution were mixed with 100 µL of thrombin stock solution. 200 µL of fibrinogen stock solution were mixed with 200 µL of HBS buffer. 400 µL of this solution (Fibrinogen + HBS) was mixed with 200 µL of CaCl_2_-thrombin solution. The gelling solution was immediately transferred in the 3D printed spheres and gelation was left to occur at room temperature for 10 min.

### FITC-dextran perfusion

At 7 days and 28 days, mice were perfused with 1 ml of 25 mg/ml FITC Dextran (150 kDa) in PBS solution containing 2% porcine gelatin and 1% copper (II) phthalocyanine via intracardiac injection as described by O’Ceallaigh *et al*.^56^. Briefly, under isolflurane anaesthesia as above, a thoracotomy is performed and the right atria is vented. A 30 gauge needle is inserted into the left ventricle and attached to warmed tyrodes buffer and the circulation is flushed at 0.5 mL per minute using a syringe pump (Harvard Apparatus) until solution form the right atria vent is clear. 50 mL of FITC-Dextran with gelatin solution is administered into the left ventricle at the same rate until the animal circulation is saturated with the dye. The gelatin facilitated vascular filling by preventing vessels collapsing, whilst the dye allowed the perfusate to be visualized. The animal is then cooled to 4 °C until the gelatin sets prior to harvest of the chambers.

### Light-sheet fluorescent microscopy (LSFM)

AVL chambers were removed from animals and fixed with zinc fixative for 24 h. Exploratory LSFM showed that the chamber and pores produced scattered images. Consequently, the outer 3D printed chamber was removed and only the tissue generated was analysed. For clearing, BABB solution was made before use by mixing 1 part benzyl alcohol with 2 parts benzyl benzoate (both from Sigma-Aldrich, St. Louis, MO, USA) in glass bottle. The sample was incubated in 1:1 mix of methanol and BABB for 10 minutes, followed by overnight incubation in 100% BABB at 4 °C in a glass vial. Samples were then held in place for LSFM by adding 0.5% agarose and allowing it to set within a 1 mL syringe. LSFM was performed on samples with a 10x objective in a Zeiss Z.1 Lightsheet Microscope.

### Whole-mount immunohistochemistry

For whole-mount immunohistochemistry the tissue was fixed by immersion in 4% paraformaldehyde (PFA), followed by 4 h incubation of the isolated sample in PFA. The AVL tissue was carefully dissected out and washed 3 × 5 minutes in PBS and 5 × 5 minute in methanol. The sample was thereafter fixed overnight at 4 °C in Dent’s fix, composed of 20% DMSO in methanol. On the second day the sample was washed 3 × 5 minutes and 3 × 1 h in PBS. Blocking was carried out overnight in 5% donkey serum and 15% DMSO in PBS at ambient temperature. Primary antibody CD31 (1:100, BD Biosciences #550274) incubation was done in blocking solution for 2 days at ambient temperature on horizontal shaker. After removal of the primary antibody the sample was rinsed 3 × 5 minutes in PBS and washed 5 × 1 hour in PBS. Secondary antibody incubation was carried out similarly to primary antibody, in the same blocking buffer for 2 days (ambient temperature, in dark). Following the secondary antibody removal the sample was again briefly rinsed with PBS and washed 3 × 1 hour in PBS. It was fixed again in 4% PFA for 1 h, washed 3 × 5 minutes and 3 × 1 h in PBS. The sample was then washed with 50% methanol in PBS for 5 minutes and in 100% methanol 4 × 10 minutes. The sample was visualized using sequential scanning mode on Leica SP5 inverted confocal microscope on 4 Well Glass Bottom µ-Slide (Ibidi Gmbh, Martinsried, Germany, Cat. No. 80427). 3D image analysis was performed using Bitplane Imaris 9 software (Bitplane AG, Zurich, Switzerland).

### Histology

The whole chamber and loop were immediately placed in zinc fixative solution for 48 h at room temperature^57^. Samples were processed in a Tissue-Tek Vacuum Infiltration Processor (Bayer Diagnostics, Newbury, UK), embedded in Paraplast Plus paraffin medium (Leica Biosystems, UK), and serial sectioned at 7 µm thickness. Sections were dewaxed in xylene and rehydrated through graded alcohol series prior to staining. Sections for immuno-staining were incubated for 20 min at room temperature with blocking serum (ImmPRESS^TM^ HRP Kit, Vector Laboratories, UK) before incubating overnight at room temperature with primary antibody: α-SMA (1:100, Abcam #5694), laminin (1:100, Abcam #11575), CD31 (1:100, BD Biosciences #550274), VEGF (1:100, Abcam #46154), BrdU (1:200, Abcam #6326) and Hsp47 (1:50, Abcam #109117). Sections for BrdU staining were exposed to 4 M HCL for 10 min and then 5 min of borate buffer prior to blocking step. Following 2 × 3 min PBS-T wash, sections were incubated for 30 min with secondary antibody (ImmPRESS^TM^ HRP Kit, anti-rabbit #MP7401 or anti-rat #MP7444, Vector, UK). Following 2 × 3 min PBS-T wash, sections were incubated for 5–10 min with DAB and nickel (DAB HRP kit, #SK4100, Vector, UK) and counterstained with nuclear fast red (Sigma-Aldrich, UK).

Sections for collagen fibre staining^58^ were stained for 1 h with 0.1% Sirius red (Sigma-Aldrich, UK) in saturated aqueous picric acid (Sigma-Aldrich, UK).

### Data analysis and statistics

For whole mount staining time points 7 days and 28 days were selected for analysis. Time-points at 4, 7, 14, 21 and 28 days were selected for general parameters, histological and immunohistochemical analysis. 7 µm thick sections were selected for either H&E, picrosirius red staining or immunohistochemistry at intervals of 112 µm. Data such as vessel counts and staining density for each chamber (N = 3) were measured and averaged from a minimum of 4 sections from the centre of each stack.

Slides were mounted and scanned with a 20x objective (Aperio Scanscope, Leica Biosystems, UK) and images were screenshot captured for analysis. Immunohistochemistry data were calculated in Fiji ImageJ (v2.0)^59^ by setting a threshold to only show DAB stained areas and then dividing by the total area to obtain a percentage of marker presence. Standard deviations (±) are given throughout. Birefringence of collagen bundles were visualized under polarized light (Axio Scope A1, Zeiss, Germany) and images were captured at 0° and 90° rotations with a Canon G9 camera and PSRemote capture software (v2.1, Breeze Systems Limited). Images were split to individual 8-bit RGB channels and a threshold was set to highlight areas of collagen in the red and the green channel. The red birefringent collagen was calculated by subtracting the total area highlighted in the red channel by the area of yellow/green birefringent collagen.

Blood vessel formation are presented as an average per mm^2^ for the number of CD31 antibody stained circles found in four 250 µm × 250 µm squares positioned 100 µm away from the outer wall of the artery and vein of the AVL. In Supplementary Fig. S4, the total number of blood vessels stained by CD31 were counted and then averaged from four sections taken from the centre of the stack for each sample. One-way ANOVA with Tukey post-hoc multiple comparisons were performed between AVL time-points to assess content weight, vessel count, collagen distribution and immunohistochemistry. Student’s t-tests were performed to compare AVL and no AVL controls at each time-point. GraphPad Prism 6 software was used to perform statistics.

### Ethical statement

All experiments were performed under the Animals (Scientific Procedures) Act 1986 and ethical standards were in accordance to UK Home Office guidelines.

## Supplementary information

Supplementary Info

Video 1

Video 2

## Data Availability

The most relevant data generated or analysed during this study are included in this published article (and its Supplementary Information Files).
