# Supplementary material for: Angiogenesis and tissue formation driven by an arteriovenous loop in the mouse
Source: Sci Rep. 2019 Jul 19;9:10478. doi: 10.1038/s41598-019-46571-4 (PMC6642172; doi:10.1038/s41598-019-46571-4)
Supplement: Supplementary file 1 — Supplementary Info [file 41598_2019_46571_MOESM1_ESM.pdf]

Angiogenesis and tissue formation driven by an arteriovenous loop in the mouse

Richard Wong[1], Roberto Donno[2 *], Christopher Y. Leon-Valdivieso[3] , Urmas Roostalu[1,4], Brian Derby[3],

Nicola Tirelli[2,5], Jason K. Wong[1,6 *]

1. Division of Cell Matrix and Regenerative Medicine, Faculty of Biology, Medicine and Health. Manchester Academic Health Science Centre. University of Manchester, Manchester, M13 9PT, UK.
2. Laboratory of Polymers and Biomaterials, Fondazione Istituto Italiano di Tecnologia, Via Morego, 30 16163 Genova, Italy
3. School of Materials, University of Manchester, Manchester, M13 9PL, UK. *Current address:* Roberval Laboratory for Mechanics, Sorbonne Universités, Université de Technologie de Compiègne, Rue du Dr. Schweitzer, 60200 Compiègne, France.
4. Gubra, Horsholm, Denmark.
5. Division of Pharmacy & Optometry, School of Health Science, University of Manchester, Manchester M13 9PT, United Kingdom
6. Department of Burns and Plastic Surgery, Manchester University Foundation Trust, Manchester Academic Health Science Centre, Wythenshawe Hospital, Southmoor Road, Manchester, M23 9LT, UK.

* Author correspondence:
Jason K. Wong (Senior Corresponding Author)
3.106 Stopford Building, Faculty of Biology, Medicine and Health, University of Manchester,
Manchester, M13 9PT, UK
Tel +44 16127 51592
jason.k.wong@manchester.ac.uk

Roberto Donno (Co-Corresponding Author)
Laboratory of Polymers and Biomaterials, Fondazione Istituto Italiano di Tecnologia, Via Morego 30,
16163 Genoa, Italy
Tel +39 010 71781 761
roberto.donno@iit.it

Supplementary Information

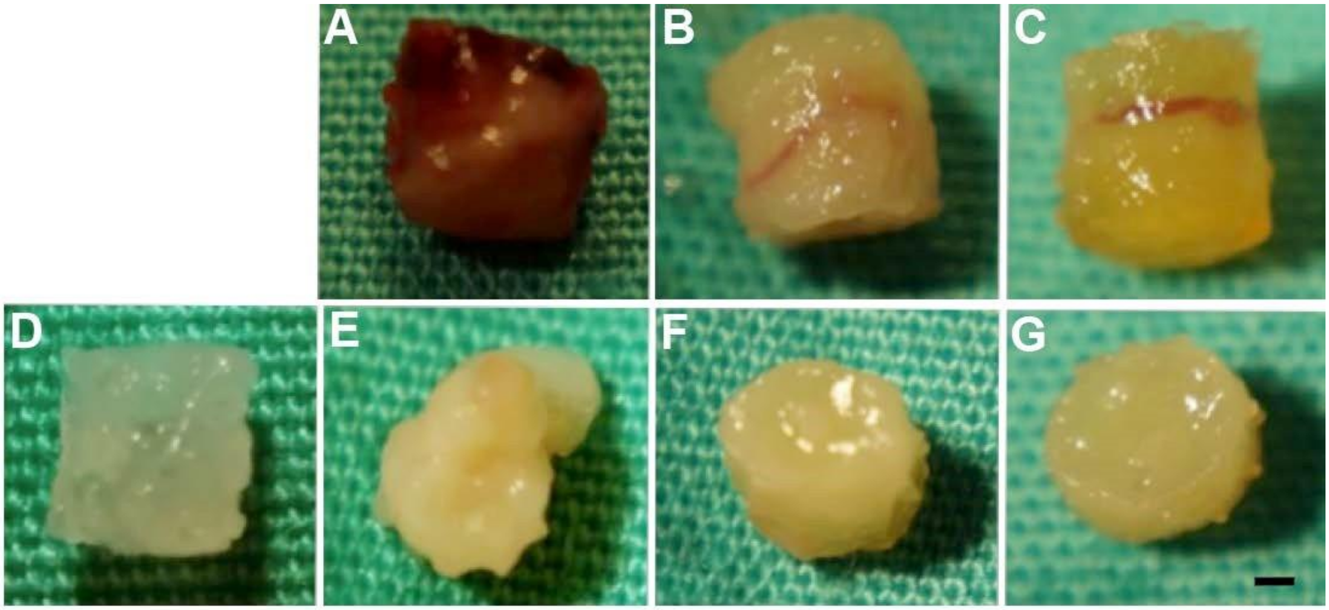

Figure S1: Appearance of the contents of chamber with AVL at (A) day 4, (B) day 21 and (C) day 28; and no AVL controls at (D) day 0, (E) day 4, (F) day 21 and (G) day 28. Scale bar = 1mm

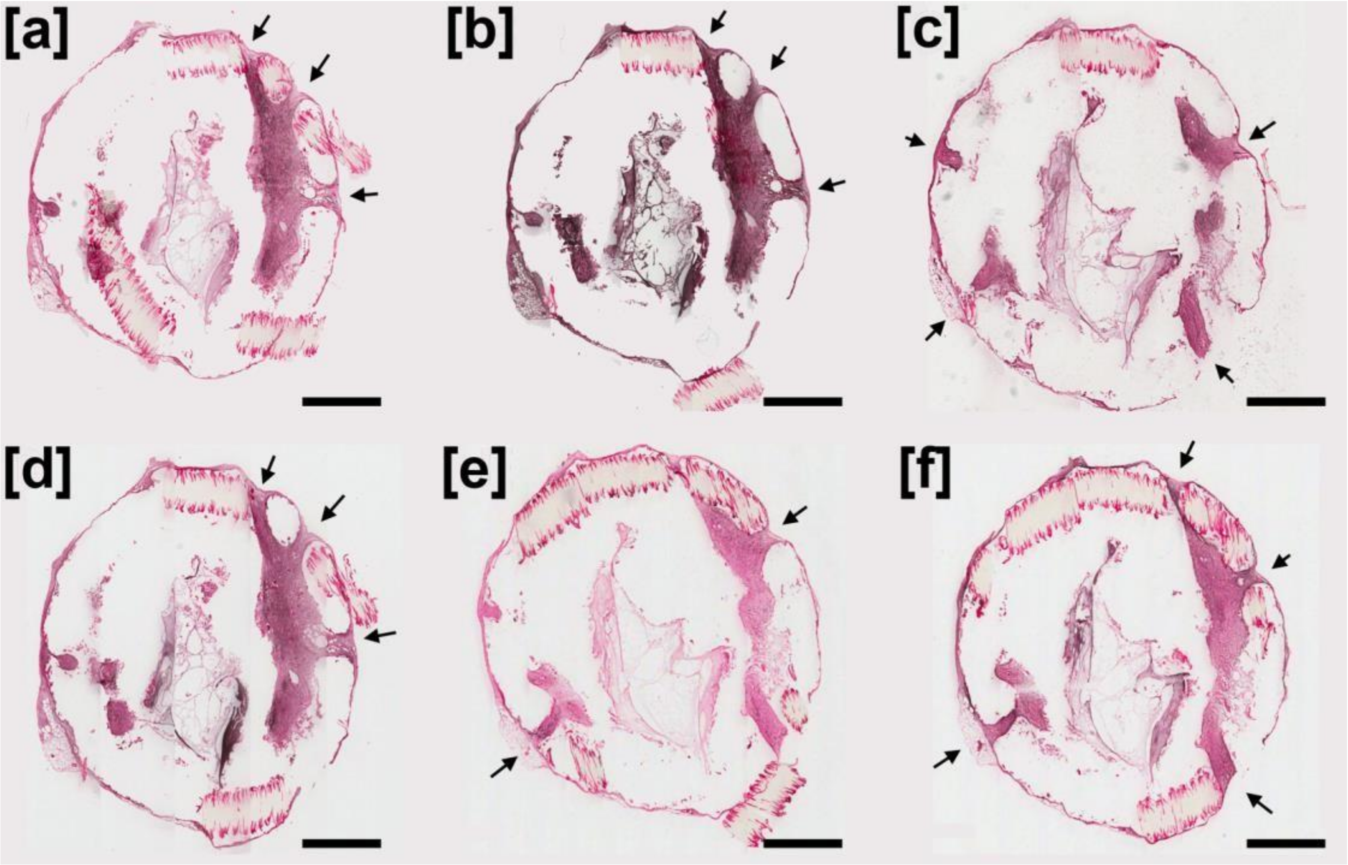

Figure S2: Immuno-staining of control chambers with no AVL at 28 days show that there is little blood vessel or tissue formation with ingress occurring via the pores (arrows). Scale bar = 1mm. (a) CD31 (b) laminin (c) α-SMA (d) VEGF (e) BrdU (f) Hsp47.

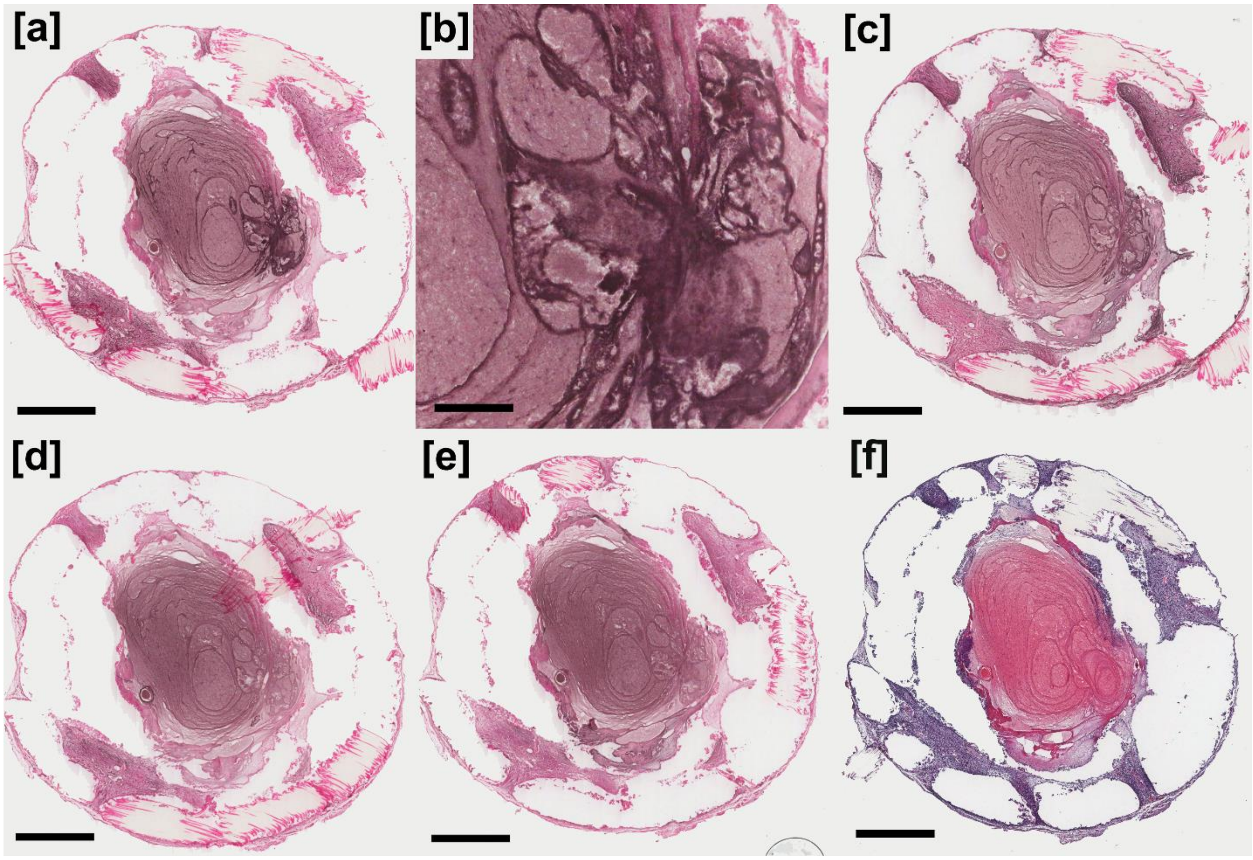

Figure S3: Immuno-staining and histology of no-flow AVL control chambers at 28 days show that there is little blood vessel or tissue formation around the ligated vessel. The vessel provides a potential source of blood and may have resulted in a haematoma appearance within the fibrin matrix. The staining and structure is different to that of the AVL samples (Figure S5). Scale bar = 1mm except (b) which is 200 µm. (a) CD31 (b) CD31 with higher magnification (c) laminin (d) α-SMA (e) VEGF (f) H&E stain.

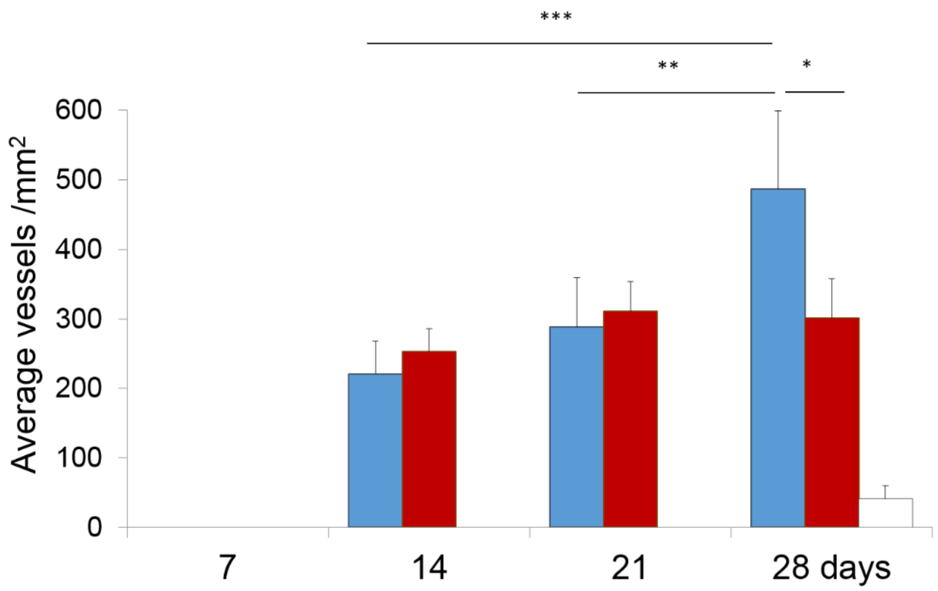

Figure S4: The graph shows the average number of blood vessels counted in 4 central sections of chambers (N=3) stained by CD31. Counts were differentiated between the venous side (blue) and the arterial side (red) of the AVL. White = no AVL control. Vessels counted show that there was a significant increase in vessels from day 14 onwards in AVL chambers. A greater number of vessels were also found from the vein side of the AVL than the arterial side on day 28. Student T-test comparisons *** $P<0.0001$, ** $P<0.001$, * $P<0.05$.

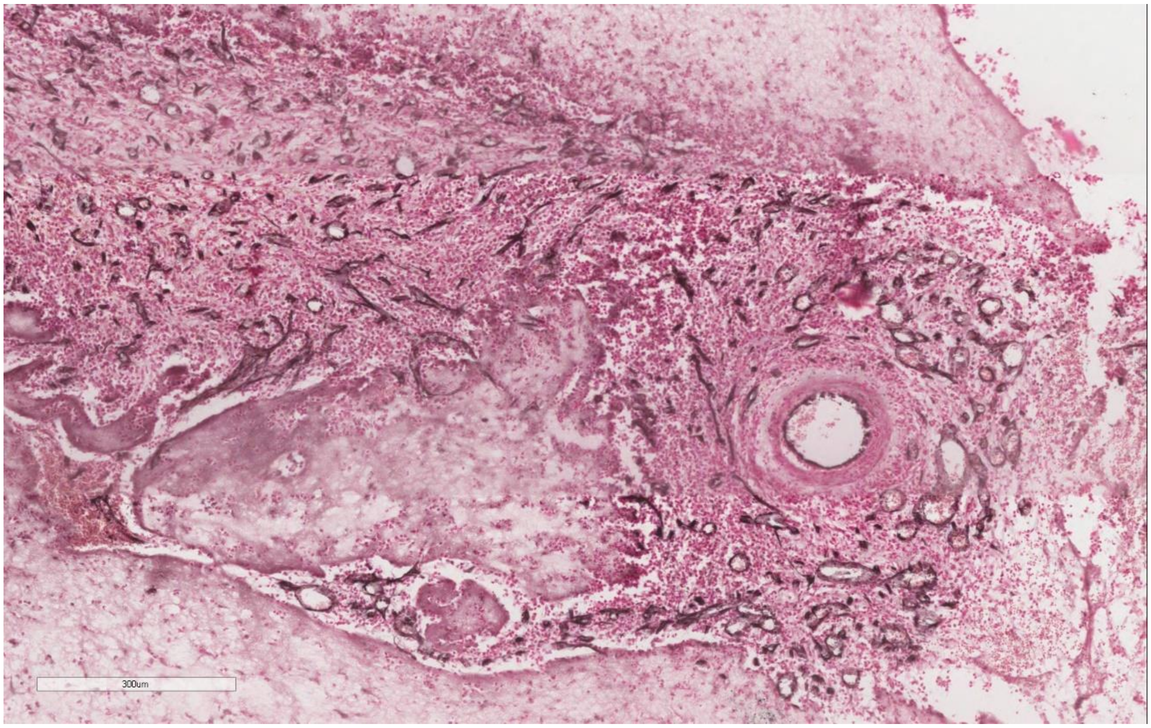

Figure S5(a) Cross-section of AVL at 14 days with CD31 staining. Vessels can be observed around the artery. Scale bar = 300 µm

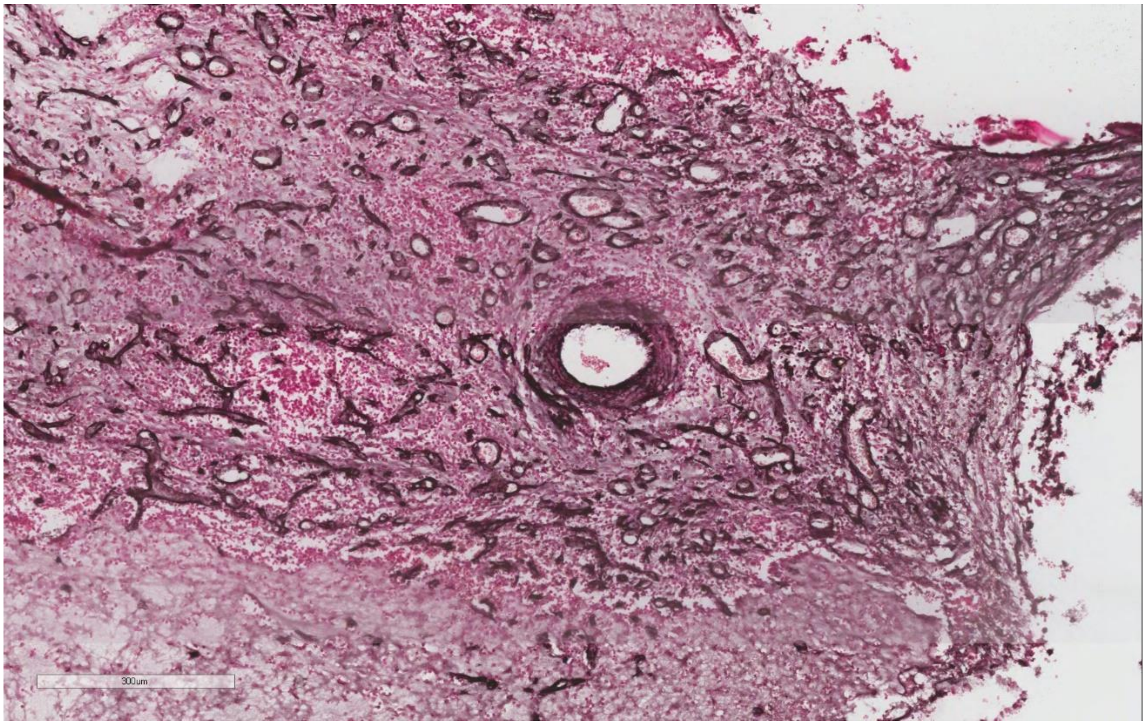

Figure S5(b) Cross-section of AVL at 14 days with laminin staining. A number of vessels can be observed around the artery. Scale bar = 300 µm

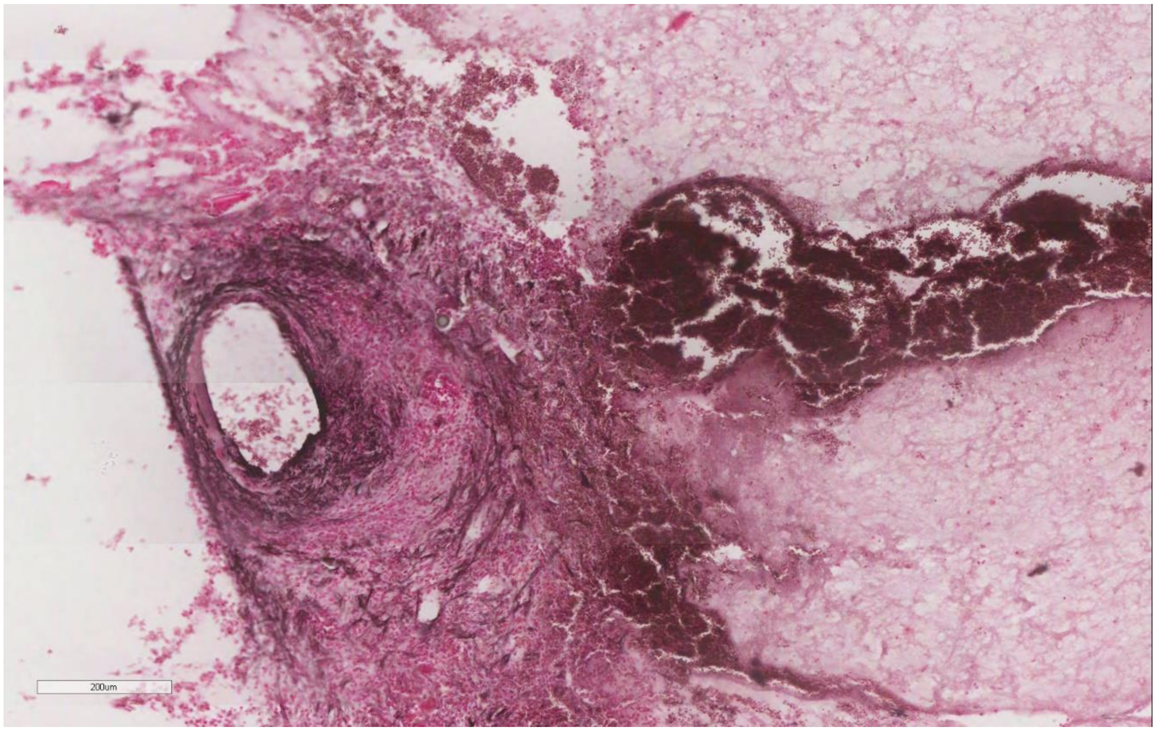

Figure S5(c) Cross-section of AVL at 14 days with α-SMA staining. The vein is visible on the left with the vessel looping from the artery (off-screen). Scale bar = 200 µm

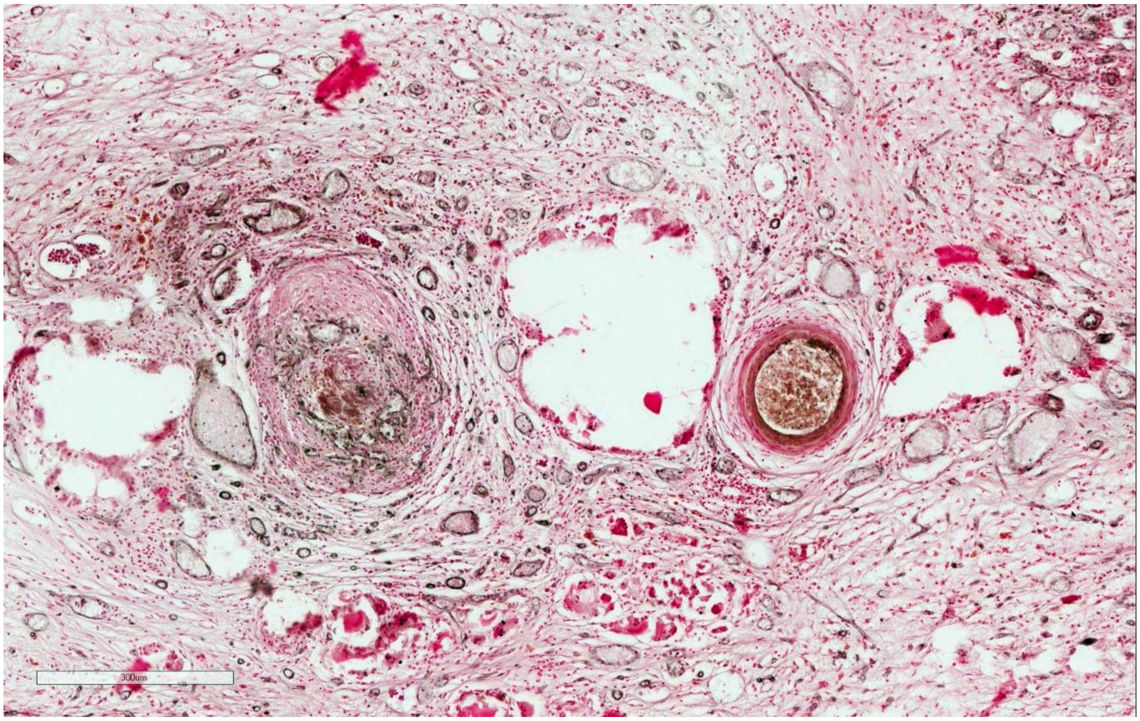

Figure S5(d) Cross-section of AVL at 28 days with CD31 staining. A greater number of vessels can be observed around the vein (left) than the artery (right). Scale bar = 300 µm

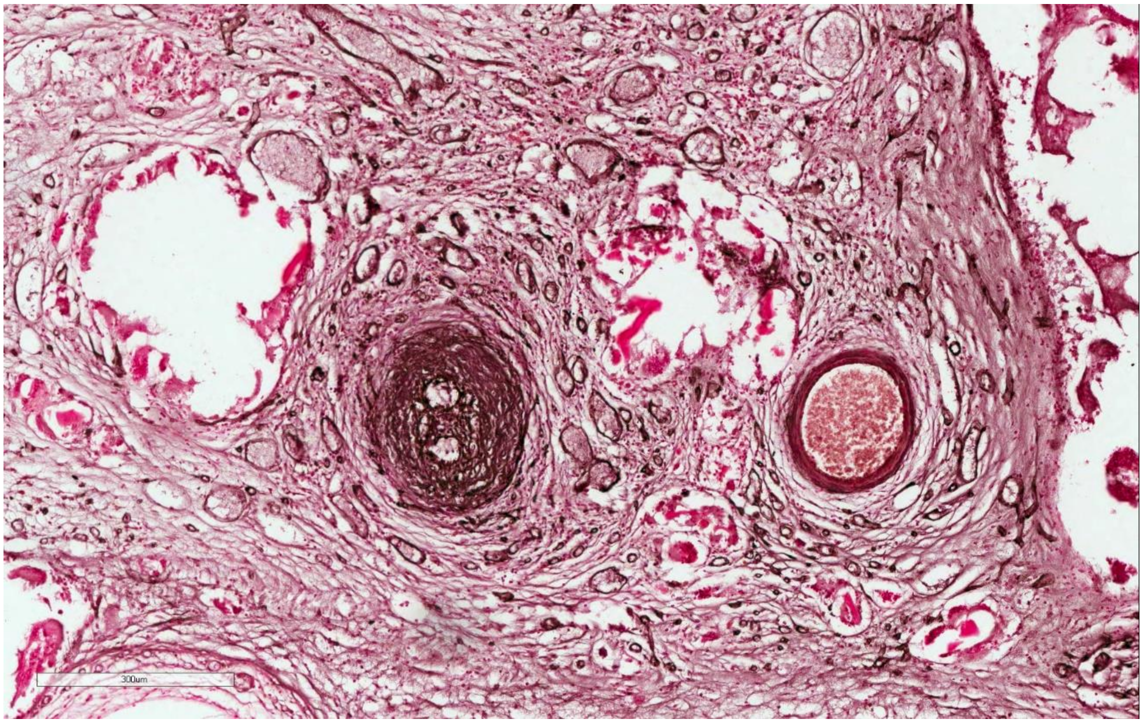

Figure S5(e) Cross-section of AVL at 28 days with laminin staining. A greater number of vessels can be observed around the vein (left) than the artery (right). Scale bar = 300 μm

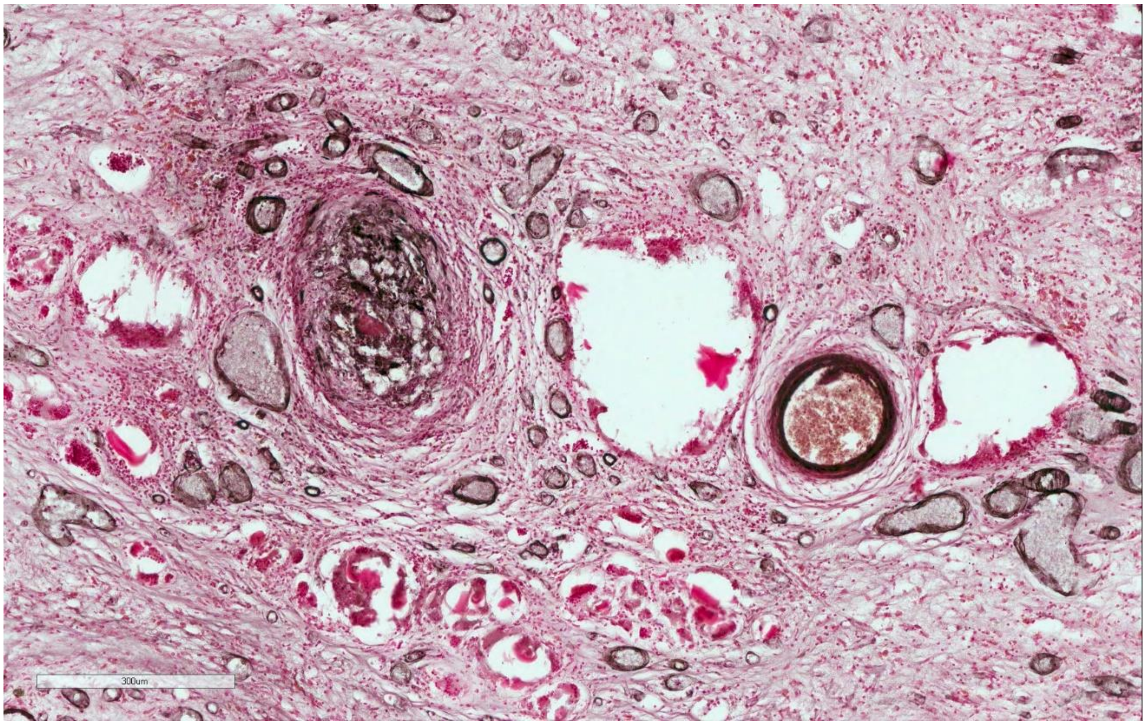

Figure S5(f) Cross-section of AVL at 28 days with α-SMA staining. A greater number of vessels can be observed around the vein (left) than the artery (right). Scale bar = 300 μm
